# An Examination of Palliative or End-of-Life Care Education in Introductory Nursing Programs across Canada

**DOI:** 10.1155/2011/907172

**Published:** 2011-10-05

**Authors:** Donna M. Wilson, Barbara L. Goodwin, Jessica A. Hewitt

**Affiliations:** ^1^Faculty of Nursing, University of Alberta, Edmonton, AB, Canada T6G 2G3; ^2^Faculty of Nursing, Helen Glass Centre, University of Manitoba, Winnipeg, MN, Canada R3T 2N2

## Abstract

An investigation was done to assess for and describe the end-of-life education provided in Canadian nursing programs to prepare students for practice. All 35 university nursing schools/faculties were surveyed in 2004; 29 (82.9%) responded. At that time, all but one routinely provided this education, with that school developing a course (implemented the next year). As compared to past surveys, this survey revealed more class time, practicum hours, and topics covered, with this content and experiences deliberately planned and placed in curriculums. A check in 2010 revealed that all of these schools were providing death education similar to that described in 2004. These findings indicate that nurse educators recognize the need for all nurses to be prepared to care for dying persons and their families. Regardless, more needs to be done to ensure novice nurses feel capable of providing end-of-life care. Death education developments will be needed as deaths increase with population aging.

## 1. Introduction

Caring for dying persons and their families has always been a major responsibility of nurses. Nurses working today can expect to care for dying persons and their families in hospitals, private residences, nursing homes, prisons, and most other places of employment [1]. This care can be very difficult to provide, in part because death is such a significant event but also because it is not always evident when a dying process is occurring. In the 1960s, the enlightened approaches of Elizabeth Kubler-Ross, Cicely Saunders, and Jeanne Quint Benoliel began to impact end-of-life care, helping to shift it from primarily cure-oriented and life-prolongation efforts to palliative or comfort-oriented care and quality-of-life-remaining efforts [2–6]. In Canada, Balfour Mount is widely considered the father of palliative care through his efforts, which include founding Montreal's Royal Victoria Hospital palliative care service in 1974/75. Today, palliative care is widely recognized as an essential healthcare service, and it has become a nursing and medical specialty in many countries [7–9]. Since 2004, the Canadian Nurses Association has had a certification program for nurses who choose to specialize in hospice/palliative care [10]. This credential, signifying an expert level of palliative care knowledge and skills, is not a prerequisite however for every nurse who provides end-of-life care. As deaths occur in many different settings and across all age groups [1, 11], every nurse must be prepared to care for dying people and their families. Palliative or end-of-life care education is thus an important component of introductory nursing programs. 

The palliative or end-of-life care education provided to nursing students in Canada and other countries has been infrequently studied, with trend and other vital information for planning and evaluation purposes thus not available. More than a decade has passed since the last published research study in 1997 by Downe-Wamboldt and Tamlyn [12] provided readily accessible information on the death education offered in introductory nursing programs to nursing students across Canada. A research investigation was subsequently designed to assess, describe, and compare palliative or end-of-life care education across Canadian undergraduate nursing programs, so as to be able to critically examine Canadian student nurse death education trends.

## 2. Background

Many research articles aimed at improving the care of terminally ill and dying persons have been published since the palliative care movement began over 30 years ago [13]. However, only two published research studies on the death education of Canadian student nurses were located [12, 14]. The more current one by Downe-Wamboldt and Tamlyn [12] provided information on nursing and medical programs across Canada and the United Kingdom. Three additional studies focused on death education for nursing students in the United Kingdom [15–17], and two studies focused on death education for student nurses in the United States [18, 19]. 

The most current investigation of Canadian education, by Downe-Wamboldt and Tamlyn [12], involved a mail questionnaire sent to 29 nursing schools, with completed surveys received from 27. All 27 had death education content, with 93% having this content integrated throughout the curriculum and 7% having a required course instead. The average total time allotted for death education per program was 24.5 classroom hours and 36.25 clinical practice hours. Lectures were the most common instructional method (70% of schools), followed by case studies, small-group discussions, and viewing audiovisual materials (59% each). Less common teaching strategies were self-directed activities (44% of schools), role-playing (30%), clinical experience (19%), and journal writing (4%). The most common educational topic was the needs of the family (88% of schools), followed by communication (82%), loss/grief (82%), and bereavement (82%). Additional common topics were spirituality (78% of schools), the role of healthcare professionals (78%), pain/symptom control (78%), dying of cancer (74%), ethical issues (70%), death anxiety (63%), cultural diversity (63%), the hospice movement (63%), dying of AIDS (59%), legal issues (56%), and body image (52%). Students were most often given tests (74% of schools) to examine their knowledge, although more than half of the schools also required students to write papers, demonstrate effective clinical practice, and/or write case studies of their experiences. Discussions (82% of schools) comprised the most common method for evaluating the student's affective domain, followed by clinical practice observation (63%), attitude measurement (26%), and death anxiety measurement (22%). 

The first investigation of death education in Canadian nursing schools, by Caty and Downe-Wamboldt [14], also employed a mail questionnaire sent to 29 nursing schools. Their study revealed 21 of the 28 responding schools (75.1%) provided death education, with 19 (90.4%) integrating it throughout the curriculum and 2 (9.5%) having a required course. Of the 7 schools that reported no mandatory death education, 4 indicated that none was provided and 3 indicated they had an elective course for students. 

These two studies show that death education had become a standard component of introductory Canadian nursing programs by the mid-1990s, as compared to the 1980s when only 3/4 schools included it. Since the 1990s, educational changes could be expected, such as new or more topics, differences in weighting of death education against other topics, evaluation methods, and teaching/learning methods. Change is also expected because context-based or problem-based learning (PBL) is now common across Canadian nursing programs [20–22]. One study has even examined the PBL approach as a method for providing death education to post-RN students [23]. The common concern with PBL of uneven learning across students was not found to be evident, as all student subjects reported fruitful discussions of personal experiences and published information [23]. Interdisciplinary courses and other teaching/learning innovations have also been tested for their effects, with some benefits determined [24–27]. These studies collectively indicate that awareness exists among nurse educators that death education should be included in introductory nursing programs.

## 3. Research Methods

In keeping with the two previous Canadian studies, a mail survey was used to gather data from nursing schools/faculties across Canada. Questions similar to those in previous surveys were included in the survey, with the draft questionnaire piloted at a local school and some questions revised. After research ethics approval was obtained from the University of Alberta's Health Research Ethics Board, all 35 schools/faculties offering introductory nurse education were surveyed in mid-2004 about the death education provided in the 2003/04 academic year. The Canadian Association of Schools of Nursing website was used to formulate this list and gain a contact name for each site, with the envelope addressed to each dean or director. All schools/faculties were situated in universities, with these offering four-year programs and with many of these universities having similar satellite programs offered in smaller universities or colleges. After a reminder letter and repeat survey were sent to nonresponders, a total of 29 completed questionnaires were received (82.9% response rate). Descriptive/comparative data analyses appropriate for the data variables were then conducted using the SPSS computer software package. These findings were later compared to those gained in 2010 from a representative at each of the same schools/faculties; teachers were asked by email communications to determine if there had been any major changes in death education since the 2003/04 year. All representatives reported (again by email) that their schools were continuing to primarily provide death education in various PBL scenarios and/or courses and through opportunities gained in a wide range of clinical venues. Few changes from the 2003/04 findings were thus identified.

## 4. Results and Discussion

### 4.1. Results

For the 2003/04 year, 28/29 (96.6%) school representatives reported that death education was a standard or mandatory curriculum component, with the one school (3.4%) not having it as such reporting that a course was in development (this course was subsequently initiated the next year). Integrating death education content throughout the program was the approach used by 16 schools (57.1%), while 12 (42.9%) schools addressed this content in a required course that all students had to take. Eight of the schools, that had content integrated throughout the curriculum, also had an elective palliative or end-of-life care course available to students. 

The 28 schools with death education present employed 5.9 teaching/learning strategies on average (5.5 median). Lectures were commonly reported (82.1% of schools), followed by small group discussions (78.6%) and case studies (67.9%). Eight (28.6%) had a palliative clinical practicum, although this was a required experience at only one school. Among these eight schools, palliative clinical practice hours ranged from 25 to 340 (179.2 mean, 151.0 median) in total. One respondent reported having an elective only practicum as there were “too many students and not enough palliative care nurses/clients.”

Four of the 28 respondents indicated that they could not tabulate instructional hours as the content was integrated throughout their PBL program, while 9 (32.1%) other respondents provided a target number of hours ranging from 4 to 215 (47.6 mean, 39 median). These instructional hours were spread over all four years (39.3%), placed entirely in the first year (35.7%), or divided across years two and three (25%).

The most frequently cited educational topics were attitudes to death and dying (92.9% of schools), communication with family and friends of the dying person (92.9%), and personal exploration in relation to death and dying (92.9%). Communication with dying patients, cultural diversity, loss/grief/bereavement, and pain and symptom management were also common topics (89.3% each), followed by the role of the nurse (85.7%), spiritual issues (82.1%), death anxiety (60.7%), legal issues (53.6%), and body image (50.0%). The average number of topics covered per school/faculty was 11.4, with 12 the median number. 

Seven respondents (25%) indicated that they did not employ any cognitive testing of students, while 21 (75%) reported evaluating cognitive knowledge through tests and/or written papers. Case study testing was also reported as occurring in 57.1% of schools/faculties, and clinical practice evaluation was reported as occurring in 66.7% of schools. Most (95.2%) of the 21 schools also evaluated the affective domain, with 95.2% using discussion-based evaluation to ensure that students were emotionally prepared to provide end-of-life care. Clinical practice evaluation was also commonly used to evaluate the student's affective domain (61.9%), in addition to attitude testing (28.6%) and death anxiety measurement (14.3%). 

Most respondents (93.1%) indicated that they personally felt death education “should be provided” to all undergraduate nursing students, with the remaining 6.9% indicating that death education “should be allotted significant attention” or “must be provided.” Most respondents (89.3%) also reported challenges in providing death education. Lack of time in the curriculum was the most frequently cited challenge (53.6%), followed by a lack of clinical placement or practice options (28.6%) and a lack of knowledgeable teachers (21.4%). One respondent indicated that his school/faculty had twice the number of students wanting to take a death education course as space allowed. Another raised the issue that if a student chose not to take the elective course and then did not care for dying patients in any practicum, that student could entirely avoid learning about death, dying, and end-of-life care. Another respondent reported highly variable student exposure to dying persons and stated the concern that not all students have multiple experiences to develop attitudes, knowledge, and skills before graduation.

### 4.2. Discussion

This survey found that palliative or end-of-life care education was a component in all but one of the surveyed introductory nursing programs in the 2003/04 year and with that school/faculty implementing a required course the next year. Although one limitation of this study is that surveys were not returned by 6 school representatives (17.1%), this study and the previous mid-1990s survey by Downe-Wamboldt and Tamlyn [12] both indicate that Canadian nursing schools typically offer death education to their nursing students. These findings are in keeping with the long-standing view among nurses that death education is essential for student nurses to prepare them for their future work [13, 28]. 

One interesting difference identified across the mid-1990s and 2003/04 study findings is that death education was reported more often in the past as being integrated throughout the curriculum, despite Canadian schools/faculties having since shifted to PBL as their primary teaching/learning approach [20–22]. It is possible that death education began to be more deliberately planned across PBL scenarios and courses, as compared to the past when lectures were the primary teaching/learning method. In the 1990s, death education would have been more dependent on a teacher recognizing its significance and having selected knowledge or skills to teach. In contrast, PBL scenarios do not require classroom tutors to have palliative care knowledge or expertise, although clinical teachers should all have at least baseline end-of-life care knowledge and skills.

In the United States, the issue of nursing teacher preparedness has been addressed by a train-the-trainer approach endorsed through the End-of-Life Nursing Education Consortium [29]. Canadian teacher training needs were recently illustrated as a concern through Brajtman, Fothergill-Bourbonnaia, Fiset, and Alain's 2009 single-site study which found only modest knowledge levels about the care of dying persons among 53 nurse educators [30]. Recently, the Canadian Hospice Palliative Care Association along with a number of Canadian nursing groups and Health Canada have collectively initiated a process to increase the amount of death education in nursing programs and also ensure its certainty there [31].

Another finding of interest, which was evident regardless of the shift to PBL as a primary instructional method, was that teaching/learning strategies were similar across the two surveys. Lectures comprised the most common teaching/learning approach by 70% and 82.1% of schools/faculties, respectively. Evaluation methods were also similar over time. Tests and written papers were the two most common methods for testing cognitive knowledge that were identified in both studies, followed by clinical practice and case study evaluations. Similarly, discussions and clinical practice observation were two consistent methods used most often to evaluate the student's affective domain. These findings demonstrate some stasis in teaching/learning and evaluation methods. This is an issue for further research and perhaps attention and change, as these teaching/learning and evaluation methods may not be the most relevant or successful. Graduating students need to be capable and feel well prepared to care for dying persons and their families. For instance, future students could be asked to: (a) write and present a scholarly paper on a chosen death/dying topic (as these activities require in-depth work and critical thinking), (b) interview family members or friends about a recent death/dying experience and then compare their findings with classmates (so they reflect on differences or similarities across people and situations), and/or (c) assist a palliative/end-of-life care research study or research proposal development process (as active learning is more effective than passive learning). 

One difference between the two studies was an increase over time in instructional hours (24.5 and 47.5 on average, respectively) and also clinical hours (36.25 and 179.2 hours on average, respectively). The increased time allotted to death education could explain the greater number of topics covered by each school/faculty in the 2003/04 year as compared to the mid-1990s. These changes suggest that educators are encouraging a more in-depth understanding of the needs of dying persons and their families. These changes could also reveal action on the enduring concern that nurses do not always feel adequately prepared to provide end-of-life care [32]. 

Although all nurse educators who responded to the 2004 survey indicated that death education should be included in introductory nursing programs, some volunteered the information that issues existed which could reduce the likelihood of every student becoming prepared to provide end-of-life care upon graduation. As indicated, these issues were essentially twofold: (a) there is a competition for time or space in very full curriculums, and (b) limited student exposure to dying persons. This space or time competition and lack of exposure to dying persons are not likely to end soon, as many important topics need to be covered in nursing curriculums, and most patients or clients are not dying. Although death education is obviously valued as it was included in all but one school/faculty (and by the next year it had been included in that school/faculty's curriculum), it is likely that death education will need to be more emphasized in the future. Population growth and population aging are beginning to double the number of persons who can be expected to die each year in Canada [1]. Currently, around 250,000 persons die annually in Canada, with 61% of deaths taking place in hospital and most of the remaining deaths occurring in nursing homes and private residences—places where nurses commonly work [1]. Although classroom preparation is clearly important for cognitive and perhaps affective foundations, clinical exposure and other real-life experiences are often essential for helping student nurses gain the knowledge, skills, and attitudes they require to provide effective end-of-life care and to be comfortable in providing that care. Creativity may be needed to expand clinical practice opportunities beyond the current heavy reliance on the two traditional training settings of hospital units and nursing homes. Additional learning opportunities, for instance, could be gained from experience with or exposure to loss/bereavement support groups, funeral home tours, code team follow-throughs, and/or shadowing organ harvesting or transplantation nurses.

## 5. Conclusions

All nurses need to be prepared to care for dying persons and their families, with introductory nursing education programs critical for this preparation. Since the mid-1990s, nurse educators in Canada appear to have continued to recognize death education as essential in introductory nursing programs. The 2004 survey findings reported here indicate that nursing student death education has been growing in significance in Canada, as more hours of classroom and clinical work are being devoted to it. However, there appear to be a number of challenges remaining to ensure that all student nurses have gained at least a basic level of theoretical and clinical preparedness upon graduation. Given the projected increase in the number of dying persons in Canada, it will be important to continue monitoring nursing student death education. Advocating for more certain death education so that all students in nursing schools/faculties gain a solid foundation for practice may be needed. In addition, research to determine the immediate and long-term outcomes of this education is recommended. Research and advocacy in other countries are also needed, as nurses worldwide must all be prepared to provide end-of-life care.
